# Increased risk of secondary bladder cancer after radiation therapy for endometrial cancer

**DOI:** 10.1038/s41598-022-05126-w

**Published:** 2022-01-20

**Authors:** Li Wen, Guansheng Zhong, Min Ren

**Affiliations:** 1 Department of Prenatal Diagnosis and Screening Center, Hangzhou Women’s Hospital (Hangzhou Maternity and Child Health Care Hospital), Hangzhou, 310008 Zhejiang People’s Republic of China; 2 Department of Breast Surgery, The First Affiliated Hospital, College of Medicine, Zhejiang University, 79 Qingchun Road, Hangzhou, 310003 Zhejiang People’s Republic of China; 3 Department of Obstetrics and Gynecology, Zhejiang Hospital, 12 Lingyin Road, Hangzhou, 310013 Zhejiang People’s Republic of China

**Keywords:** Cancer epidemiology, Gynaecological cancer, Urological cancer

## Abstract

To investigate the effect of radiation therapy (RT) after endometrial cancer (EC) diagnosis on the risk of occurring secondary bladder cancer (SBC) as well as on the survival outcome of those patients who suffered with SBC. Data was extracted from the Surveillance, Epidemiology, and End Results database between 1973 and 2015. Chi-squared test was utilized to compare clinicopathological characteristics among different groups. The Fine and Gray’s competing risk model was utilized to assess cumulative incidence and risk of occurring SBC in EC survivors. The Kaplan–Meier method and the Cox regression model were used for survival analysis. As a result, a total of 108,060 EC patients were included, among which 37,118 (34.3%) patients received RT while others did not. The incidence of SBC was 1.31%, 1.76% and 0.96% among patients who received prior brachytherapy, external-beam radiotherapy (EBRT) and others, respectively. Both of the EBRT (standardized incidence ratio (SIR) = 2.24, 95% CI [1.94–2.58]) and brachytherapy (SIR = 1.76, 95% CI [1.44–2.13]) group had a higher incidence of SBC than the general population in USA. The competing risk analysis demonstrated that receiving EBRT (HR = 1.97, 95% CI [1.64–2.36]) or brachytherapy (HR = 1.46, 95% CI [1.14–1.87]) were all independent risk factors for developing SBC. A survival detriment was only observed in SBC patients who received prior EBRT after EC diagnosis, but not for brachytherapy, when compared with those who did not undergo RT. Additionally, there were no significant survival differences between primary bladder cancer and SBC with or without prior RT history. Patients who underwent RT after EC had an increased risk of developing bladder cancer as secondary primary cancer. The prognosis of these SBC patients varied depending on types of RT that received after EC diagnosis.

## Introduction

Endometrial cancer (EC) represents the most commonly diagnosed gynecologic malignancy as well as the fourth most common malignancy in females, accounting for ~ 7% of all newly diagnosed cancer in the USA^1^. A latest cancer statistical report estimates that approximately 63,230 new EC cases are expected to be diagnosed in the USA, in 2018^1^. Due to the relatively early symptom of vaginal bleeding and successful treatment, the majority of EC patients have a favorable clinical outcome, with a 5-years overall survival rate of 80 to 85%^2^. Consequently, the number of long-term survivors of EC has increased rapidly, and these patients are at increased risk of occurring secondary primary cancer in comparison with the general population^3,4^. A combination of factors, such as genetic susceptibility, unhealthy lifestyle as well as previous EC-specific treatment could be possible reason^5^.

Postoperative radiation therapy (RT) has been routinely utilized for EC patients thought to be at high risk of recurrence^2,6^. With a goal of treating possible micro-metastasis disease in pelvic lymph-node regions, radiotherapy can be delivered to the pelvis externally, as vaginal brachytherapy, or as a combination^7^. Several studies reported that a prior RT may contribute to the development of various second primary malignancies^8,9^. A previous report made by Gonzales et al. revealed that approximately 8% of the secondary solid cancer could be associated with RT^10^. Nevertheless, Wiltink et al. reported that no increased risk of second malignancy after RT was observed in a meta-analysis pooling > 2500 patients with pelvic cancers from randomized TME (Total Mesorectal Excision)^11^, PPRTEC-1 (Post Operative Radiation Therapy in Endometrial Carcinoma 1)^2^, and PORTEC-2^12^ trials^13^. Moreover, a decreased risk of developing prostate cancer after pelvic RT for rectal cancer was also observed in a previously published study^14^. Hence, it is still controversial for the increased risk of developing secondary primary malignancy after RT.

Specifically, the bladder is within the irradiation field when RT is conducted for endometrial cancer which located in the uterus. Considering the early and late toxicity associated with RT, the current study aimed to study the impact of RT on the risk of developing secondary bladder cancer (SBC) in EC survivors as well as on the prognosis of patients suffered with SBC, by utilizing the Surveillance, Epidemiology, and End Results (SEER) database. Our investigation might provide an important clue for future RT selection, patients counseling and prevention strategies among EC survivors at increased risk of developing SBC.

## Materials and methods

### Database and case selection

We performed a retrospective research by utilizing the custom SEER database [Incidence- SEER 9 Regs Custom Data (with additional treatment fields), Nov 2017 Sub (1973–2015)]. The SEER program, a database established by the National Cancer Institute of the U.S., collected data of cancer patients that accounts for about approximately 28% of the U.S. population^15^. The SEER*Stat software (version 8.3.8, National Cancer Institute, Washington, USA) was used to access the data from SEER database. Patients who were diagnosed with EC (site code C54.0-54.3, C54.8, C54.9, and C55.9) between 1973 and 2015 were extracted from the database. Only patients who had undergone endometrial cancer specific surgery and had endometrial cancer as their first malignancy were eligible. Exclusion criteria was listed as follows: (1) patients younger than 18 years old at diagnosis; (2) patients with unknown information of survival time; (3) patients with unknown information of race; (4) patients whose diagnosis was made at autopsy or based on a death certificate.

**Figure 1:**
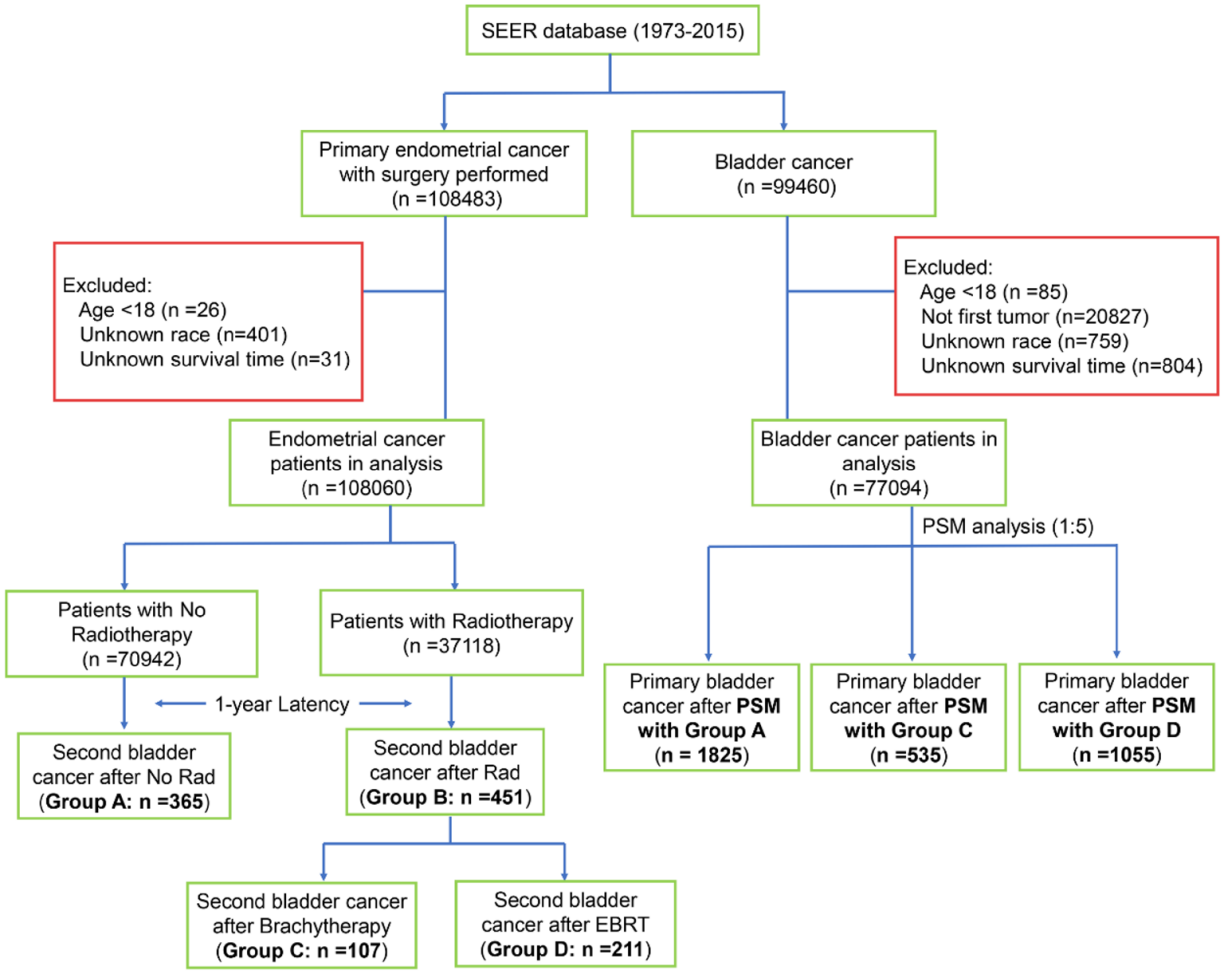
The flowchart of patient selection.

Eligible endometrial cancer patients were grouped into two subgroups based on whether they received RT or not. Subsequent SBC were eligible when it occurred more than 12 months after EC diagnosis. Patients in the no RT cohort who developed SBC were classified into group A, while those in the RT cohort who developed SBC were classified into group B. Based on the RT modality, group B was further divided into patients who received brachytherapy (Group C) and those received external-beam radiation therapy (EBRT) (Group D). Moreover, patients were also extracted from SEER database if diagnosed with a first primary bladder cancer (PBC) from 1973 to 2015. In order to reduce possible selection bias for survival comparison, three cohorts of female PBC patients were matched respectively for group A, C and D by using the propensity score matching (PSM) method with a ratio of 5:1. The detailed flowchart for the patient’s selection was shown in Fig. 1.

All methods were performed in accordance with the relevant guidelines and regulations. The SEER database is an open database. Data released from the SEER database do not require informed patient consent, because cancer is a reportable disease in every state of the United States. The present study complied with the 1964 Helsinki Declaration and its later amendments or comparable ethical standards.

### Covariates and outcomes

Multiple variables were included in this study, including demographic characteristics (age, race and year of diagnose), disease characteristics (stage and histologic grade), and treatment modalities (radiotherapy and chemotherapy). Specially, races, including American Indians, Asians, AK Natives and Pacific Islanders, were classified into other races. Continuous variable, such as age and years at diagnosis, was transformed into categorical variable. According to the “radiation recode” in SEER database, radiotherapy for endometrial cancer were classified into EBRT and brachytherapy (radioactive implants). Patients who received combination of two or more RT modalities were excluded in this study. The primary outcome was to evaluate the risk of occurring SBC among patients who had not received EC specific RT as well as among those who had received different RT modalities. The secondary outcome was to assess the impact of EC specific RT on overall survival (OS) and bladder cancer specific survival (BCSS) of those SBC patients and compared it to matched PBC patients.

### Statistical analysis

Demographic and clinical characteristics between different cohorts were summarized by descriptive statistics and compared by using the Pearson’s Chi-square test. The standardized incidence ratios (SIRs) for SBC after EC diagnosis were defined by calculating the ratio of observed-to-expected (O/E) incidence, which represents change of the risk of developing SBC after EC diagnosis in comparison with the general US population. The SIR analysis was performed by using the SIR tools in SEER program software (SEER*Stat 8.3.6). In order to assess the risk of SBC dynamically, the SIRs were stratified by latency time after EC diagnosis, age and year at EC diagnosis.

Fine and Gray competing risk analysis was utilized to evaluate the risk of developing SBC after EC diagnosis. Specifically, SBC occurrence was considered as an event and all non-SBC caused deaths were defined as competing events. Cumulative incidence curve for SBC occurrence was plotted and compared by Gray’s test^16^. Besides, univariate and multivariable Fine and Gray competing risk regression models and Cox proportional hazards regression model were also built to analyze BCSS and OS, respectively. By means of a backward selection method, variable with *P* value of < 0.05 in univariable analysis was included in multivariable analyses. The Kaplan–Meier curves were plotted for the OS and BCSS between different cohorts, and the Log Rank test was utilized for comparison between curves.

Descriptive statistic, and the Cox analysis were conducted by utilizing the SPSS 24.0 (IBM Corp). The Fine and Gray competing risk analysis, cumulative incidence curve and Kaplan–Meier curves were performed and plotted by utilizing the R software (version 4.0.0). We defined a 2-sided *P* value of < 0.05 as statistically significant.

## Results

### Patient characteristics

A total of 108,060 EC patients were finally extracted from the SEER database, among which 37,118 (34.3%) patients had received RT, and 70,942 (65.7%) patients had not received RT. Compared with patients who did not undergo RT, patients who received RT presented with older age, earlier diagnosis, poorer histologic differentiation, higher rate of white race and regional stage. More patients in RT group received chemotherapy than no RT group. Additionally, among RT group, patients who underwent EBRT had a higher rate of grade 3–4, regional and distant stage (*P* < 0.001). The detailed information for clinicopathological features among these different groups were listed in Table 1. After one year latency since EC diagnosis, a total of 451 survivors in RT cohort and 365 in no RT cohort were diagnosed with a SBC at the end of the follow-up. Moreover, among those SBC patients in RT cohort, 211 patients received EBRT and 107 patients received brachytherapy.

**Table 1:** Baseline characteristics of endometrial cancer patients (N = 108,060).

Characteristics	Total patients		*P* values	Patients received radiotherapy		*P* values
	No radiotherapy N = 70,942 (%)	Radiotherapy N = 37,118 (%)		Brachytherapy N = 10,801 (%)	EBRT N = 26,317 (%)	
Age (years), mean (SD)	61.0 (12.0)	63.0 (10.7)	< 0.001	62.4 (10.4)	63.2 (10.8)	< 0.001
**Age (years)**			< 0.001			< 0.001
< 60	32,244 (45.5)	13,523 (36.4)		4252 (39.4)	9271 (35.2)	
60–79	34,004 (47.9)	21,500 (57.9)		5981 (55.4)	15,519 (59.0)	
≥ 80	4694 (6.6)	2095 (5.6)		568 (5.3)	1527 (5.8)	
**Year of diagnose**			< 0.001			< 0.001
1973–1989	21,174 (29.8)	17,039 (45.9)		4967 (46.0)	12,072 (45.9)	
1990–2004	26,307 (37.1)	10,903 (29.4)		1622 (15.0)	9281 (35.3)	
2005–2015	23,461 (33.1)	9176 (24.7)		4212 (39.0)	4964 (18.9)	
**Race**			< 0.001			< 0.001
White	61,083 (86.1)	32,826 (88.4)		9849 (91.2)	22,977 (87.3)	
Black	3896 (5.5)	2177 (5.9)		551 (5.1)	1626 (6.2)	
Others^a^	5963 (8.4)	2115 (5.7)		401 (3.7)	1714 (6.5)	
**Grade**			< 0.001			< 0.001
Grade 1–2	51,149 (72.1)	19,828 (53.4)		6214 (57.5)	13,614 (51.7)	
Grade 3	8199 (11.6)	10,512 (28.3)		2236 (20.7)	8276 (31.4)	
Unknown	11,594 (16.3)	6778 (18.3)		2351 (21.8)	4427 (16.8)	
**Stage**			< 0.001			< 0.001
Localized	30,966 (43.6)	7640 (20.6)		3648 (33.8)	3992 (15.2)	
Regional	4004 (5.6)	5983 (16.1)		1374 (12.7)	4609 (17.5)	
Distant	1459 (2.1)	590 (1.6)		75 (0.7)	515 (2.0)	
Unknown	34,513 (48.6)	22,905 (61.7)		5704 (52.8)	17,201 (65.4)	
**Chemotherapy**			< 0.001			0.185
No	66,440 (93.7)	32,095 (86.5)		9379 (86.8)	22,716 (86.3)	
Yes	4502 (6.3)	5023 (13.5)		1422 (13.2)	3601 (13.7)	

EBRT, External beam radiotherapy.

^a^Including Asian and American Indians.

### Cumulative incidence of SBC in EC survivors

**Figure 2:**
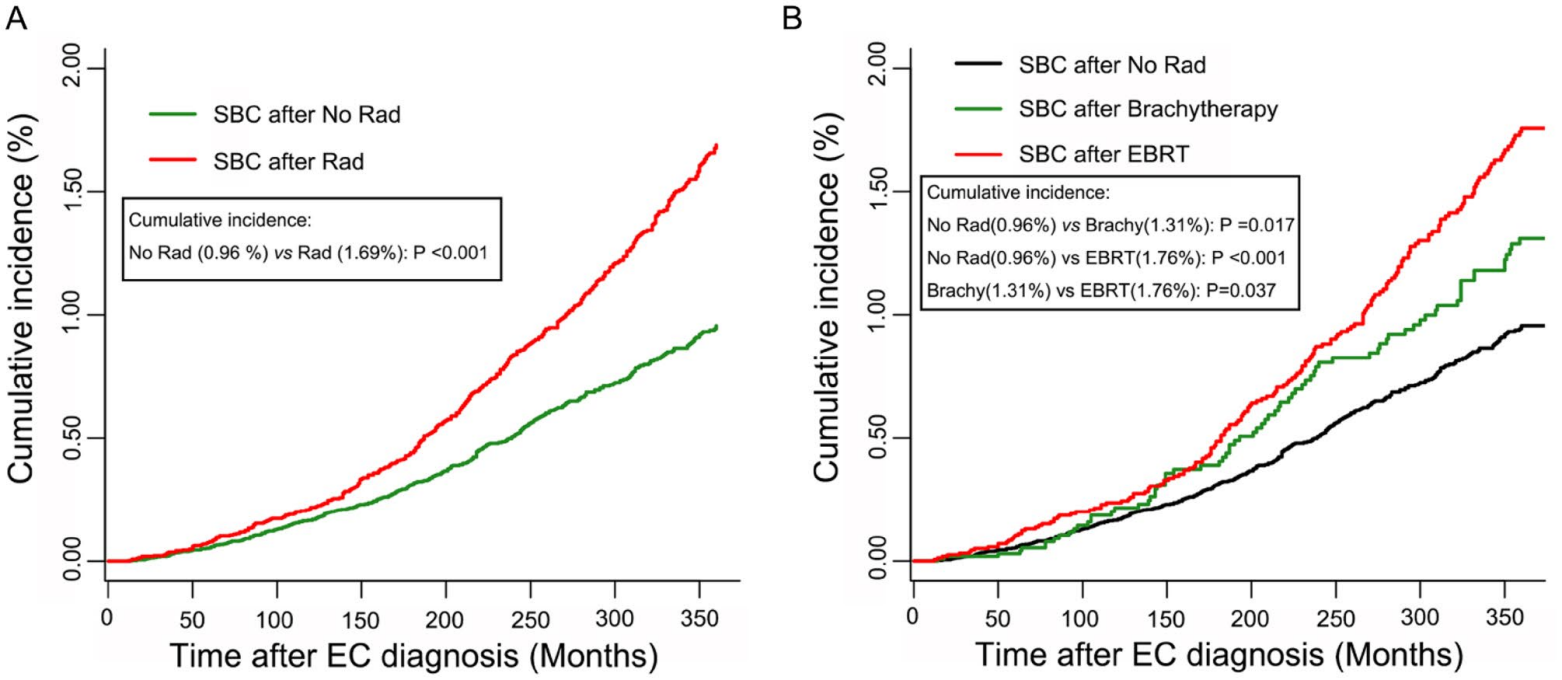
The cumulative incidence of secondary bladder cancer (SBC) in EC survivors. (**A**) Comparison of cumulative incidence between patients received radiotherapy or not; (**B**) Comparison of cumulative incidence among patients received different RT modality. *P* values were calculated with the Gray test. Rad, Radiotherapy; SBC, secondary bladder cancer; EC, endometrial cancer; EBRT, external-beam radiotherapy.

The cumulative incidence of SBC in EC patients who underwent RT or not was compared in this study. As shown in Fig. 2A, EC patients who received any RT were more likely to develop SBC than patients who did not receive RT, with cumulative incidence being 1.69% and 0.96% (*P* < 0.001), respectively. Moreover, when RT group were subdivided into brachytherapy and EBRT categories, the cumulative incidence between RT and no RT groups remained significant (No RT vs brachytherapy: *P* = 0.017; No RT vs EBRT: *P* < 0.001) (Fig. 2B).

The SIRs of SBC were also calculated in EC survivors with different radiotherapy modalities. In comparison with the US general population, the incidence of SBC was dramatically increased both in brachytherapy (SIR = 1.76, 95% CI [1.44–2.13]) and EBRT (SIR = 2.24, 95% CI [1.94–2.58]) group (Table 2). Nevertheless, a similar incidence of SBC was found among EC survivors who did not undergo RT (SIR = 0.99, 95% CI [0.89–1.10]). In sub-analyses, SIR for SBC was stratified by latency time after EC diagnosis, year and age at EC diagnosis. As shown in Table 2, no significant incidence change was observed for patients who did not undergo RT in all subgroups in comparison with the US general population. In latency-SIR sub-analyses, EC patients undergone brachytherapy had significantly increased incidence of SBC only after more than 5 years of follow-up. However, in comparison with the US general population, the incidence of SBC was significantly increased after 1 year of follow-up after received EBRT. In sub-analyses of age or year of EC diagnosis, dramatical increase in the incidence of SBC was observed in almost all subgroups of EC survivors regardless of receiving brachytherapy or EBRT (Table 2).

**Table 2:** Standardized incidence ratio of secondary bladder cancer in EC patients.

Variables	No radiotherapy		Brachytherapy		EBRT	
	SIR (95% CI)	*P* value	SIR (95% CI)	*P* value	SIR (95% CI)	*P* value
Total patients	0.99 (0.89–1.10)	ns	1.76 (1.44–2.13)	< 0.05	2.24 (1.94–2.58)	< 0.05
**Latency, months**		ns				
12–59	1.21 (0.97–1.49)	ns	0.95 (0.48–1.71)	ns	1.62 (1.41–1.85)	< 0.05
60–119	1.13 (0.92–1.39)	ns	1.31 (1.12–1.73)	< 0.05	1.83 (1.27–2.56)	< 0.05
≥ 120	0.85 (0.72–0.99)	< 0.05	2.09 (1.65–2.62)	< 0.05	2.79 (2.34–3.30)	< 0.05
**Age at EC diagnosis**						
20–59	1.54 (0.99–2.28)	ns	2.79 (1.02–6.07)	< 0.05	3.76 (1.80–6.92)	< 0.05
60–79	0.93 (0.80–1.08)	ns	1.41 (1.04–1.87)	< 0.05	1.99 (1.61–2.43)	< 0.05
≥ 80	1.01 (0.85–1.10)	ns	2.17 (1.62–2.84)	< 0.05	2.46 (1.98–3.02)	< 0.05
**Year at EC diagnosis**						
1973–1989	0.84 (0.59–1.16)	ns	1.73 (1.11–2.58)	< 0.05	2.05 (1.41–2.87)	< 0.05
1990–2004	1.02 (0.87–1.19)	ns	1.40 (0.99–1.94)	ns	2.06 (1.66–2.54)	< 0.05
2005–2015	1.00 (0.85–1.17)	ns	2.24 (1.64–3.00)	< 0.05	2.62 (2.06–3.28)	< 0.05

SIR, standardized incidence ratio; EC, endometrial cancer; EBRT, external-beam radiotherapy; ns, no significance.

### Effect of RT on risk for developing SBC in EC survivors

To further investigate effects of RT on SBC risk, Fine and Gray competing risk regression analysis were conducted (Table 3). In univariate analysis, we demonstrated that both of the brachytherapy (HR = 1.46, 95% CI [1.14–1.87]) and EBRT (HR = 1.97, 95% CI [1.64–2.36]) were significantly related with elevated risk of developing SBC. Additionally, the increased risk of developing SBC was also observed in elderly patients and in patients with white race compared with other race, such as Asian and American Indians. Moreover, the multivariate analysis further confirmed that both of the brachytherapy (HR = 1.43, 95% CI [1.12–1.84]) and EBRT (HR = 1.89, 95% CI [1.58–2.28]) were independent risk factors for SBC occurrence in EC survivors (Table 3).

**Table 3:** Univariable and multivariable competing risk analysis of risk of developing SBC in EC survivors.

Variables	Univariable analysis		Multivariable analysis	
	HR (95% CI)	*P* value	HR (95% CI)	*P* value
**Age**				
< 60	Reference		Reference	
60–79	1.64 (1.37–1.96)	< .001	1.53 (1.27–1.83)	< .001
≥ 80	1.47 (1.05–2.07)	0.026	1.43 (1.02–2.02)	0.039
**Diagnosis year**				
1973–1989	Reference			
1990–2004	1.03 (0.87–1.23)	0.690		
2005–2015	0.99 (0.74–1.34)	0.980		
**Race**				
White	Reference		Reference	
Black	0.74 (0.47–1.15)	0.180	0.74 (0.47–1.15)	0.180
Others^a^	0.38 (0.22–0.67)	< .001	0.43 (0.25–0.74)	0.002
Unknown	0.64 (0.90–4.56)	0.660	0.75 (0.11–5.39)	0.780
**Grade**				
1–2	Reference			
3	1.15 (0.93–1.44)	0.200		
Unknown	1.02 (0.82–1.28)	0.850		
**SEER stage**				
Localized	Reference			
Regional	1.05 (0.70–1.59)	0.800		
Distant	0.40 (0.15–1.10)	0.075		
Unknown	0.88 (0.71–1.09)	0.230		
**Chemotherapy**				
No	Reference			
Yes	1.00 (0.70–1.43)	0.980		
**Radiotherapy**				
No	Reference		Reference	
Brachytherapy	1.46 (1.14–1.87)	0.003	1.43 (1.12–1.84)	0.005
EBRT	1.97 (1.64–2.36)	< .001	1.89 (1.58–2.28)	< 0.001

CI, confidence interval; HR, Hazard ratio; EBRT, external-beam radiotherapy; EC, endometrial cancer.

^a^Including Asian and American Indians.

### Effect of RT on survival of SBC in EC survivors

**Figure 3:**
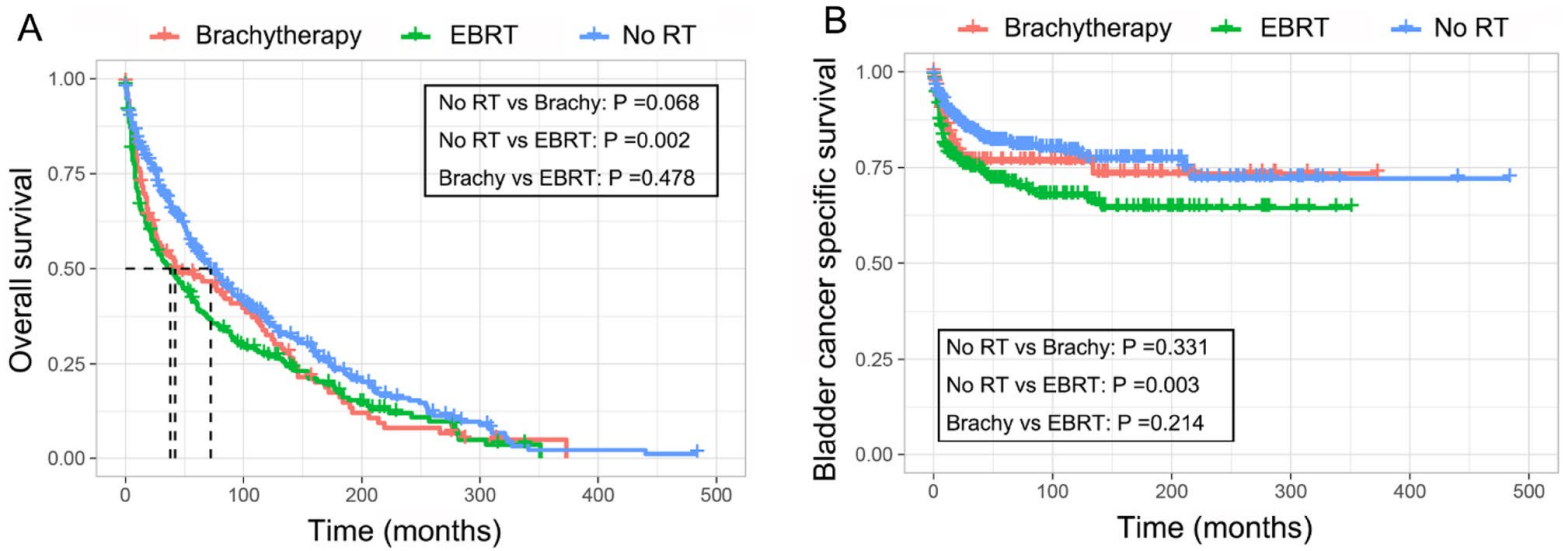
Kaplan–Meier curve of OS (**A**) and BCSS (**B**) in SBC patients received different RT modality after EC diagnosis. OS, overall survival; BCSS, bladder cancer-specific survival; EBRT, external-beam radiotherapy.

Both of the OS and BCSS between SBC patients treated with RT or not were compared in this study. As shown in Fig. 3, the Kaplan–Meier curves indicated that patients who received prior EBRT had significant inferior OS (*P* = 0.002) and BCSS (*P* = 0.003) when compared with patients who did not receive RT. However, our results also showed that brachytherapy had no significant effect on OS and BCSS of SBC patients when compared with no RT patients. Additionally, no significant survival difference was also observed between brachytherapy group and EBRT group. In order to further understand the effect of RT on prognosis of EC survivors who developed SBC, we then performed univariate and multivariate Cox and competing risk analysis for OS and BCSS, respectively. The multivariate analysis, as shown in Table 4, further demonstrated that a prior EC-specific EBRT was an independent prognostic factor both for OS (HR = 1.35, 95% CI [1.12–1.64]) and BCSS (HR = 1.51, 95% CI [1.05–2.18]) of EC survivors who developed SBC. Moreover, our result also indicated that variables including age, diagnosis year, histologic grade, tumor stage, and surgery could significantly affect SBC patients’ OS or BCSS. The detail information for the survival analysis was shown in Table 4.

**Table 4:** Univariate and multivariate analysis for OS and BCSS of EC survivors who had SBC.

Characteristic	OS				BCSS			
	Univariate analysis		Multivariate analysis		Univariate analysis		Multivariate analysis	
	HR (95% CI)	*P*	HR (95% CI)	*P*	HR (95% CI)	*P*	HR (95% CI)	*P*
**Age**								
< 60	1.0 (Ref)		1.0 (Ref)		1.0 (Ref)		1.0 (Ref)	
60–79	1.91 (1.27–2.87)	0.002	1.71 (1.13–2.58)	0.011	1.51 (0.65–3.49)	0.338	1.26 (0.54–2.97)	0.597
≥ 80	4.70 (3.09–7.15)	< .001	3.72 (2.42–5.72)	< .001	3.61 (1.57–8.31)	0.003	2.24 (0.95–5.28)	0.066
**Diagnosis year**								
1973–1989	1.0 (Ref)		1.0 (Ref)		1.0 (Ref)		1.0 (Ref)	
1990–2004	0.83 (0.67–1.02)	0.077	0.95 (0.75–1.21)	0.683	0.68 (0.48–0.96)	0.030	0.70 (0.46–1.08)	0.104
2005–2015	0.56 (0.43–0.73)	< .001	0.74 (0.52–1.05)	0.094	0.33 (0.19–0.59)	< .001	0.39 (0.19–0.81)	0.012
**Race**								
White	1.0 (Ref)		NA	NA	1.0 (Ref)		NA	NA
Black	0.94 (0.59–1.51)	0.804			0.53 (0.17–1.65)	0.269		
Others^a^	1.16 (0.64–2.11)	0.625			0.28 (0.04–2.01)	0.206		
**Grade**								
1/2	1.0 (Ref)		1.0 (Ref)		1.0 (Ref)		1.0 (Ref)	
3/4	2.34 (1.94–2.82)	< .001	1.86 (1.52–2.27)	< .001	5.74 (3.72–8.87)	< .001	4.06 (2.57–6.41)	< .001
Unknown	1.86 (1.40–2.45)	< .001	1.51 (1.12–2.02)	0.006	3.71 (2.05–6.71)	< .001	2.37 (1.27–4.44)	0.007
**SEER stage**								
Localized	1.0 (Ref)		1.0 (Ref)		1.0 (Ref)		1.0 (Ref)	
Regional	2.83 (1.87–4.29)	< .001	2.12 (1.38–3.24)	0.001	5.91 (3.42–10.2)	< .001	3.58 (2.04–6.29)	< .001
Distant	9.29 (6.06–14.3)	< .001	5.79 (3.70–9.05)	< .001	18.8 (11.2–31.6)	< .001	10.9 (6.08–19.5)	< .001
Unknown	0.82 (0.68–1.00)	0.051	1.15 (0.89–1.49)	0.301	0.99 (0.68–1.47)	0.993	1.83 (1.10–3.06)	0.020
**Surgery**								
No	1.0 (Ref)		1.0 (Ref)		1.0 (Ref)		1.0 (Ref)	
Yes	0.58 (0.42–0.81)	0.001	0.62 (0.44–0.89)	0.009	0.33 (0.20–0.54)	< .001	0.44 (0.25–0.77)	0.004
**Chemotherapy**								
No	1.0 (Ref)		1.0 (Ref)		1.0 (Ref)		1.0 (Ref)	
Yes	1.35 (1.03–1.78)	0.030	1.08 (0.81–1.45)	0.598	1.71 (1.11–2.63)	0.015	0.98 (0.60–1.58)	0.922
**Bladder specific radiotherapy**								
No radiation	1.0 (Ref)		1.0 (Ref)		1.0 (Ref)		1.0 (Ref)	
Radiation	1.69 (1.69–1.19)	0.003	1.13 (0.78–1.65)	0.517	2.22 (1.30–3.78)	0.004	1.21 (0.67–2.20)	0.527
**Prior EC specific radiotherapy**								
No	1.0 (Ref)		1.0 (Ref)		1.0 (Ref)		1.0 (Ref)	
Brachytherapy	1.25 (0.98–1.58)	0.074	1.19 (0.93–1.54)	0.171	1.25 (0.78–2.01)	0.353	1.01 (0.62–1.64)	0.976
EBRT	1.35 (1.12–1.64)	0.002	1.31 (1.08–1.59)	0.007	1.69 (1.19–2.40)	0.003	1.51 (1.05–2.18)	0.026

HR, hazard ratio; Ref, reference category; CI, confidence interval; EBRT, external-beam radiotherapy; OS, overall survival; BCSS, bladder cancer-specific survival; SBC, secondary bladder cancer; EC, endometrial cancer.

^a^Others including Asian and American Indians.

**Figure 4:**
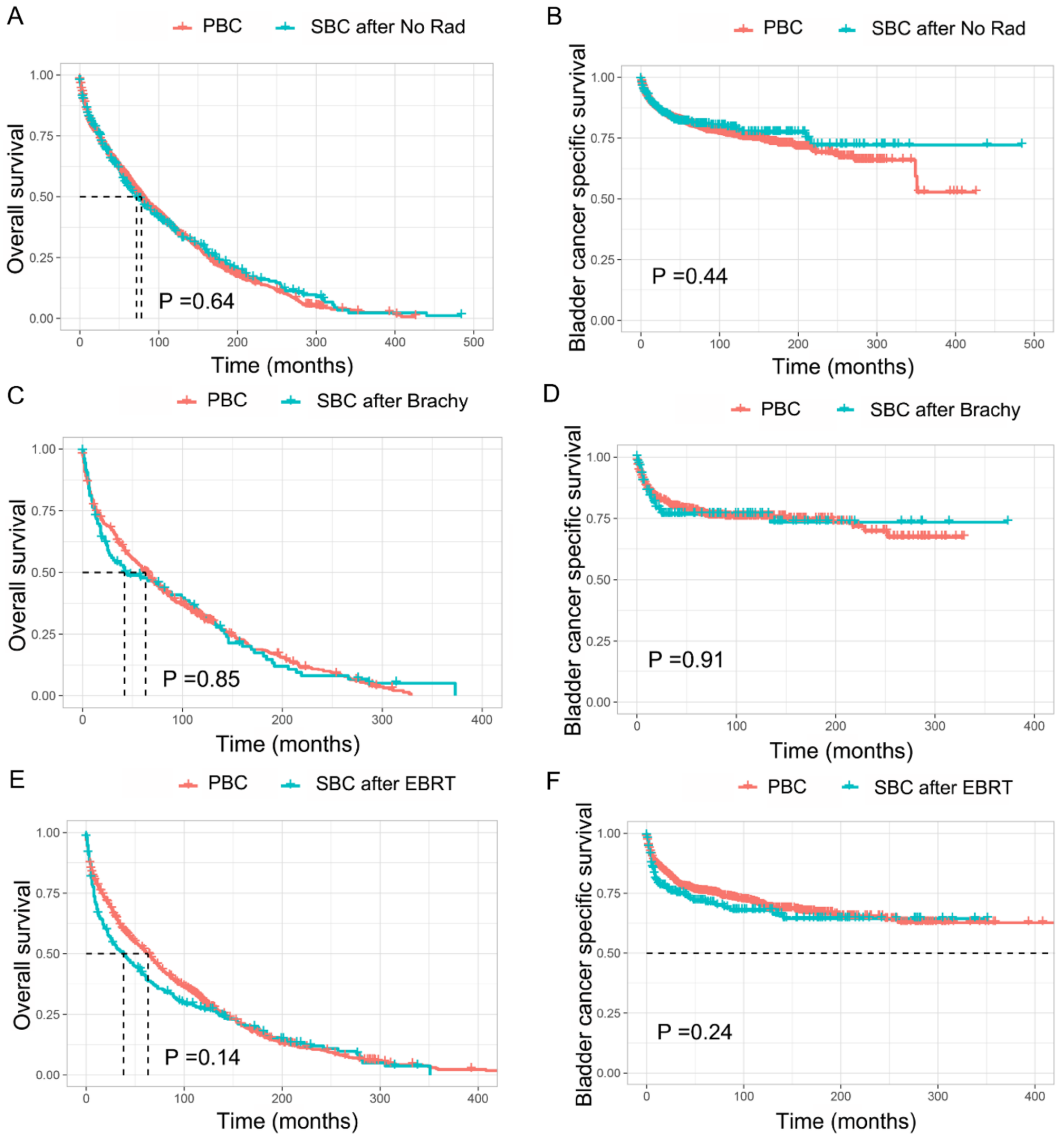
Comparison of survival between PBC patients and SBC patients received different RT modality after EC diagnosis. (**A**) OS between PBC and SBC after no radiotherapy; (**B**) BCSS between PBC and SBC after no radiotherapy; (**C**) OS between PBC and SBC after brachytherapy; (**D**) BCSS between PBC and SBC after brachytherapy; (**E**) OS between PBC and SBC after EBRT; (**F**) BCSS between PBC and SBC after EBRT; OS, overall survival; BCSS, bladder cancer-specific survival; EBRT, external-beam radiotherapy. Rad, Radiotherapy; PBC, primary bladder cancer; SBC, secondary bladder cancer; EC, endometrial cancer.

In order to understand if bladder cancer is different after RT, the Pearson’s Chi-square test was used to compare the SBC patients who received RT or not. However, no significant difference was observed (Supplementary Table 1). Subsequently, by using the PSM method, three cohorts of primary bladder cancer patients were matched separately for SBC patients who previously treated with brachytherapy, EBRT or no RT after EC diagnosis. After adjusted for propensity score, all features were well balanced between matched PBC and SBC patients after EC diagnosis (Supplementary Table 2–4). As shown in Fig. 4, three cohorts of matched PBC patients all had similar OS and BCSS as compared with SBC patients who previously treated with brachytherapy, EBRT or no RT after EC diagnosis.

## Discussion

The present study concentrated on evaluating the effect of a prior RT on the risk of occurring SBC in EC survivors as well as on the prognosis of subsequent SBC. Our data showed that the cumulative incidence of SBC in EC survivors who underwent brachytherapy or EBRT was dramatically higher than patients who did not undergo RT. Both of the brachytherapy and EBRT were demonstrated as independent risk factors for developing SBC in EC survivors. A survival detriment was only observed in SBC patients who underwent prior EBRT, but not for brachytherapy after EC diagnosis, as compared with patients who did not undergo prior RT.

Several previous studies have evaluated the risk for developing SBC in patients received pelvic RT for their pelvic cancer, with varying results. A publication by Wiltink et al. included a total of 2500 EC or RC patients pooling data from three randomized trial and reported that patients who underwent brachytherapy or EBRT had no increased risk of SBC occurrence as compared with patients received surgery alone^13^. A randomized trial reported by Onsrud et al. randomly assigned 568 patients with stage I endometrial cancer to either vaginal radium brachytherapy (VBT) followed by EBRT or VBT alone. An increased risk (HR = 1.42, 95% CI [1.01–2.00]) of secondary cancer was observed in EBRT group as compared with the control group. Importantly, the proportion of SBC was higher in the EBRT group (3.7%) than in the control group (2.6%)^17^. However, the small sample size, with only 13 SBC observed, caused limited statistical power for the conclusion. Wang et al. assessed the risk of developing secondary cancer in rectal cancer (RC) survivors received pre- or postoperative RT by using the Taiwan’s National Health Insurance Research Database^18^. Their result showed an increased risk for SBC occurrence among patients who underwent postoperative RT but nor for those underwent preoperative RT.

When death was not considered as a competing event, the probability of developing SBC may overestimate due to the number of patients who died before experiencing SBC. Hence, the Fine-Gray competing risk model was utilized in our study to analyze the risk of SBC occurrence. Our result demonstrated that EC survivors who underwent postoperative brachytherapy or EBRT all had an increased risk of developing SBC in comparison with patients received no RT. This could be attributable to the fact that the typical radiation fields of both EBRT and vaginal cuff brachytherapy for EC have included a portion of bladder. Our result also indicated that EBRT would result in higher risk of developing SBC than brachytherapy, which could be explained by a dose effect of RT. A similar dose-dependent association were reported for SBC after pelvic RT for cervical cancer^19,20^.

Additionally, the SIR analysis in our study showed a significantly high probability of developing a SBC among EC survivor who received prior brachytherapy or EBRT, as compared with the US general population. This result echoed previous studies concentrating on evaluating the risk of second primary malignancy in EC survivors^21,22^. However, our data also confirmed that EC survivors who did not undergo RT had similar incidence risk of developing SBC in comparison with the US general population, which further implied that SBC may be induced by RT treatment. In SIR sub-analyses stratified by latency time after EC diagnosis, no obvious increase of SBC incidence was observed in the early follow-up after brachytherapy but not for EBRT. We also found that the SBC incidence increased with the prolongation of follow-up time after EC diagnosis, especially after a latency of over 10 years. Currently, the primary objective of surveillance in EC survivors is to detect recurrence or metastasis within 3–5 years of follow-up^23^. However, our data suggested that EC survivors who received prior RT would benefit from long-term detection of SBC. Regarding to the effect of age on the risk of SBC, our data showed that the younger EC survivors who underwent RT had the highest risk of occurring SBC as compared with the elderly patients. A possible explanation might be that a relative longer life expectancy would increase the risk of SBC occurrence. During the period from 1973 to 2015, RT has shifted toward hypofractionation and more precise radiotherapy target area formulation, which may reduce the extra radiation exposure for the surrounding tissues around the tumor. Generally speaking, the advancement of RT technology may reduce the SBC occurrence. However, our study observed that the SBC incidence increased from 1973–2004 to 2005–2015 both in brachytherapy and EBRT group, but not in no RT group. Although it is difficult to explain, this tendency indicate that this effect was also due to the RT. We suspect that an increasing number of EC survivors would result in an increased SBC potential because more cancer patients had been cured with the advancement of RT technology.

In order to further study the impact of EC-specific RT on prognosis of subsequent SBC, survival analyses were conducted to compare OS and BCSS of SBC after RT with those who did not undergo prior RT. Our result demonstrated that patients who received prior EBRT had significant inferior survival as compared with patients who did not undergo prior RT. We suspect that a SBC after EBRT might have different biological behavior due to induction of distinct tumorigenic signaling pathways after radiation exposure. Moreover, no survival difference was observed between brachytherapy group and no RT group, implying that brachytherapy had less radiation damage on adjacent bladder than EBRT. By means of PSM method, we also demonstrated no significant survival differences between PBC and SBC with or without prior RT history. This result is supported by many previous studies demonstrated that a history of prior cancer has no impact on survival of various cancers^24–26^.

Several limitations exist in this study. Firstly, it is not entirely clear whether the radiotherapy was given only adjuvant or encompass also treatment in a recurrence setting. Secondly, the risk factors for endometrial and bladder cancer are difference and some predisposing factors, such as smoking history, lifestyle and genetic susceptibility, are unavailable in SEER database. Thirdly, it is uncapable to determine whether SBC were EC recurrences in the SEER database. Fourthly, selection bias is inherent in our study due to the intrinsic weaknesses of retrospective databases.

In conclusion, the current study confirmed that patients who underwent RT for a primary endometrial cancer had an increased risk for developing bladder cancer as a secondary primary cancer. A prior EC-specific EBRT, but not brachytherapy, had an adversely impact on the survival of SBC patients. There was no significant survival difference between PBC and SBC with or without prior RT history.

## Supplementary Information

Supplementary Information.

## Data Availability

The data analyzed in this study were extracted from publicly available datasets, which can be found here: https://seer.cancer.gov/.
