# Supplementary material for: Increased risk of secondary bladder cancer after radiation therapy for endometrial cancer
Source: Sci Rep. 2022 Jan 20;12:1032. doi: 10.1038/s41598-022-05126-w (PMC8776857; doi:10.1038/s41598-022-05126-w)
Supplement: Supplementary file 1 — Supplementary Information. [file 41598_2022_5126_MOESM1_ESM.docx]

Table S1. Baseline characteristics of endometrial cancer patients who developed SBC after received RT or not.

| Characteristics | SBC after no RT  N=365 (%) | SBC after RT  N= 451 (%) | P values |
| --- | --- | --- | --- |
| Age (years), mean (SD) | 74.0 (9.6) | 75.9 (8.7) | 0.013 |
| Year of diagnose |  |  | 0.077 |
| 1973-1989 | 53 (14.5) | 91 (20.2) |  |
| 1990-2004 | 160 (43.8) | 196 (43.5) |  |
| 2005-2015 | 152 (41.6) | 164 (36.4) |  |
| Race |  |  | 0.466 |
| White | 339 (92.9) | 427 (94.7) |  |
| Black | 14 (3.8) | 15 (3.3) |  |
| Others^a^ | 12 (3.3) | 9 (2.0) |  |
| Grade |  |  | 0.255 |
| Grade 1-2 | 161 (44.1) | 196 (43.5) |  |
| Grade 3-4 | 163 (44.7) | 187 (41.5) |  |
| Unknown | 41 (11.2) | 68 (15.1) |  |
| Histological type |  |  |  |
| Transitional cell carcinoma | 324 (88.8) | 384 (85.1) | 0.420 |
| Squamous cell carcinoma | 4 (1.1) | 6 (1.3) |  |
| Other types | 37 (10.1) | 61 (13.5) |  |
| Stage |  |  | 0.896 |
| Localized | 208 (57.0) | 250 (55.4) |  |
| Regional | 10 (2.7) | 11 (2.5) |  |
| Distant | 8 (2.2) | 13 (2.9) |  |
| Unknown | 139 (38.1) | 177 (39.2) |  |
| Bladder Surgery |  |  | 0.357 |
| No | 23 (6.3) | 36 (8.0) |  |
| Yes | 342 (93.7) | 415 (92.0) |  |
| Bladder Radiotherapy |  |  | 0.697 |
| No | 204 (96.7) | 1028 (97.4) |  |
| Yes | 7 (3.3) | 27 (2.6) |  |
| Bladder Chemotherapy |  |  | 0.133 |
| No | 342 (93.7) | 433 (96.0) |  |
| Yes | 23 (6.3) | 18 (4.0) |  |

Abbreviation: SBC, secondary bladder cancer; RT, Radiotherapy

^a^ including Asian and American Indians

Table S2. Baseline characteristics of EC patients who developed SBC after received brachytherapy and matched PBC patients.

| Characteristics | SBC after Brachytherapy  N=107 (%) | PBC  N= 535(%) | P values |
| --- | --- | --- | --- |
| Age (years), mean (SD) | 76.3 (8.5) | 76.6 (7.97) | 0.594 |
| Year of diagnose |  |  | 0.934 |
| 1973-1989 | 27 (25.2) | 130 (24.3) |  |
| 1990-2004 | 38 (35.5) | 200 (37.3) |  |
| 2005-2015 | 42 (39.3) | 205 (38.4) |  |
| Race |  |  | 0.869 |
| White | 104 (97.2) | 519 (97.0) |  |
| Black | 2 (1.9) | 8 (1.5) |  |
| Others^a^ | 1 (0.9) | 8 (1.5) |  |
| Grade |  |  | 0.560 |
| Grade 1-2 | 56 (52.3) | 250 (46.7) |  |
| Grade 3-4 | 40 (37.4) | 227 (42.5) |  |
| Unknown | 11 (10.3) | 58 (10.8) |  |
| Stage |  |  | 0.993 |
| Localized | 47 (43.9) | 250 (46.8) |  |
| Regional | 4 (3.7) | 23 (4.2) |  |
| Distant | 7 (6.5) | 34 (6.4) |  |
| Unknown | 49 (45.8) | 228 (42.6) |  |
| Bladder Surgery |  |  | 0.946 |
| No | 8 (7.5) | 39 (7.3) |  |
| Yes | 99 (92.5) | 496 (92.7) |  |
| Bladder Radiotherapy |  |  | 0.058 |
| . No | 98 (91.6) | 513 (95.9) |  |
| Yes | 9 (8.4) | 22 (4.1) |  |
| Bladder Chemotherapy |  |  | 0.635 |
| No | 98 (91.6) | 497 (92.9) |  |
| Yes | 9 (8.4) | 38 (7.1) |  |

Abbreviation: SBC, secondary bladder cancer; PBC, primary bladder cancer.

^a^ including Asian and American Indians

Table S3. Baseline characteristics of endometrial cancer patients who developed SBC after received no radiotherapy and matched PBC patients.

| Characteristics | SBC after no radiotherapy  N=365 (%) | PBC  N= 1825 (%) | P values |
| --- | --- | --- | --- |
| Age (years), mean (SD) | 74.0 (9.6) | 74.2 (9.7) | 0.589 |
| Year of diagnose |  |  | 0.691 |
| 1973-1989 | 53 (14.5) | 291 (15.9) |  |
| 1990-2004 | 160 (43.8) | 762 (41.8) |  |
| 2005-2015 | 152 (41.6) | 772 (42.3) |  |
| Race |  |  | 0.482 |
| White | 339 (92.9) | 1723 (94.4) |  |
| Black | 14 (3.8) | 59 (3.2) |  |
| Others^a^ | 12 (3.3) | 43 (2.4) |  |
| Grade |  |  | 0.606 |
| Grade 1-2 | 161 (44.1) | 789 (43.2) |  |
| Grade 3-4 | 163 (44.7) | 796 (43.6) |  |
| Unknown | 41 (11.2) | 240 (13.2) |  |
| Stage |  |  | 0.784 |
| Localized | 208 (57.0) | 1024 (56.1) |  |
| Regional | 10 (2.7) | 68 (3.7) |  |
| Distant | 8 (2.2) | 47 (2.6) |  |
| Unknown | 139 (38.1) | 686 (37.6) |  |
| Bladder Surgery |  |  | 0.550 |
| No | 23 (6.3) | 131 (7.2) |  |
| Yes | 342 (93.7) | 1694 (92.8) |  |
| Bladder Radiotherapy |  |  | 0.201 |
| No | 342 (93.7) | 1739 (95.3) |  |
| Yes | 23 (6.3) | 86 (4.7) |  |
| Bladder Chemotherapy |  |  | 0.261 |
| No | 311 (85.2) | 1511 (82.8) |  |
| Yes | 54 (14.8) | 314 (17.2) |  |

Abbreviation: SBC, secondary bladder cancer; PBC, primary bladder cancer.

^a^ including Asian and American Indians

Table S4. Baseline characteristics of endometrial cancer patients who developed SBC after received EBRT and matched PBC patients.

| Characteristics | SBC after EBRT  N=211 (%) | PBC  N= 1055 (%) | P values |
| --- | --- | --- | --- |
| Age (years), mean (SD) | 74.0 (9.6) | 74.3 (9.6) | 0.921 |
| Year of diagnose |  |  | 0.313 |
| 1973-1989 | 42 (19.9) | 237 (22.5) |  |
| 1990-2004 | 94 (44.5) | 411 (39.0) |  |
| 2005-2015 | 75 (35.5) | 407 (38.5) |  |
| Race |  |  | 0.578 |
| White | 199 (94.3) | 1011 (95.8) |  |
| Black | 9 (4.3) | 35 (3.3) |  |
| Others^a^ | 3 (1.4) | 9 (0.9) |  |
| Grade |  |  | 0.762 |
| Grade 1-2 | 81 (38.4) | 424 (40.2) |  |
| Grade 3-4 | 100 (47.4) | 471 (44.6) |  |
| Unknown | 30 (14.2) | 160 (15.2) |  |
| Stage |  |  | 0.613 |
| Localized | 94 (44.5) | 497 (47.1) |  |
| Regional | 13 (6.2) | 57 (5.4) |  |
| Distant | 13 (6.2) | 45 (4.3) |  |
| Unknown | 91 (43.1) | 456 (43.2) |  |
| Bladder Surgery |  |  | 0.707 |
| No | 15 (7.1) | 83 (7.9) |  |
| Yes | 196 (92.9) | 972 (92.1) |  |
| Bladder Radiotherapy |  |  | 0.097 |
| No | 204 (96.7) | 1038 (98.4) |  |
| Yes | 7 (3.3) | 17 (1.6) |  |
| Bladder Chemotherapy |  |  | 0.199 |
| No | 192 (91.0) | 986 (93.5) |  |
| Yes | 19 (9.0) | 69 (6.5) |  |

Abbreviation: SBC, secondary bladder cancer; EBRT, External beam radiotherapy; PBC, primary bladder cancer.

^a^ including Asian and American Indians
